# The Growth and N Retention of Two Annual Desert Plants Varied Under Different Nitrogen Deposition Rates

**DOI:** 10.3389/fpls.2019.00356

**Published:** 2019-03-26

**Authors:** Xiaoqing Cui, Ping Yue, Wenchao Wu, Yanming Gong, Kaihui Li, Tom Misselbrook, Keith Goulding, Xuejun Liu

**Affiliations:** ^1^Key Laboratory of Plant-Soil Interactions of the Ministry of Education, Beijing Key Laboratory of Farmland Soil Pollution Prevention and Remediation, College of Resources and Environmental Sciences, China Agricultural University, Beijing, China; ^2^Sino-France Institute of Earth Systems Science, Laboratory for Earth Surface Processes, College of Urban and Environmental Sciences, Peking University, Beijing, China; ^3^State Key Laboratory of Desert and Oasis Ecology, Xinjiang Institute of Ecology and Geography, Chinese Academy of Sciences, Ürümqi, China; ^4^Urat Desert-Grassland Research Station, Northwest Institute of Eco-Environment and Resources, Chinese Academy of Sciences, Lanzhou, China; ^5^College of Life Sciences, University of the Chinese Academy of Sciences, Beijing, China; ^6^Department of Sustainable Soils and Grassland Systems, Rothamsted Research, Devon, United Kingdom; ^7^Department of Sustainable Soils and Grassland Systems, Rothamsted Research, Harpenden, United Kingdom

**Keywords:** desert plants, annual, ephemeral, ^15^N tracer, biomass, ^15^N retention, N deposition

## Abstract

Nitrogen (N) partitioning between plant and soil pools is closely related to biomass accumulation and allocation, and is of great importance for quantifying the biomass dynamics and N fluxes of ecosystems, especially in low N-availability desert ecosystems. However, partitioning can differ among species even when growing in the same habitat. To better understand the variation of plant biomass allocation and N retention within ephemeral and annual species we studied the responses of *Malcolmia* *Africana* (an ephemeral) and *Salsola affinis* (an annual) to N addition, including plant growth, N retention by the plant and soil, and N lost to the environment using ^15^N (double-labeled ^15^NH_4_^15^NO_3_ (5.16% abundance) added at 0, 0.8, 1.6, 3.2, and 6.4 g pot^-1^, equivalent to 0, 15, 30, 60, and 120 kg N ha^-1^) in a pot experiment. Higher N addition (N120) inhibited plant growth and biomass accumulation of the ephemeral but not the annual. In addition, the aboveground:belowground partitioning of N (the R:S ratio) of the ephemeral decreased with increasing N addition, but that of the annual increased. The N input corresponding to maximum biomass and ^15^N retention of the ephemeral was significantly less than that of the annual. The aboveground and belowground retention of N in the ephemeral were significantly less than those of the annual, except at low N rates. The average plant–soil system recovery of added ^15^N by the ephemeral was 70%, significantly higher than that of the annual with an average of 50%. Although the whole plant–soil ^15^N recovery of this desert ecosystem decreased with increasing N deposition, our results suggested that it may vary with species composition and community change under future climate and elevated N deposition.

## Introduction

Nitrogen (N) limitation of net primary productivity in terrestrial ecosystems is globally distributed, including desert ecosystems (LeBauer and Treseder, 2008; Yahdjian et al., 2011). A meta-analysis showed that N addition could significantly increase aboveground net productivity of water-limited, i.e., desert and semi-arid ecosystems (Yahdjian et al., 2011). However, the critical loads for N deposition in desert ecosystems are thought to be lower than those for other ecosystems due to their N-poor status and low biomass (Fenn et al., 2010). It has been suggested that desert ecosystems are particularly susceptible to small increases in N inputs due to the sensitivity of desert plants to N. However, different desert plant species or plant-functional types have different growth responses to N. In the Mojave Desert, for example, increased N input from atmospheric deposition or from other sources at a rate of 3.2 g N m^-2^ yr^-1^ decreased the biomass of native annual plants but increased the density and biomass of alien annual plants (Brooks, 2003). In a pot and field experiment in the Gurbantuggut Desert, ephemeral biomass significantly increased and its allocation to roots significantly decreased with N application compared with annuals (Zhou et al., 2011, 2014). In response to chronic N addition in a temperate desert ecosystem, biomass change was non-linear and N rate-dependent: low and intermediate levels of N increased biomass but high levels (24 g N m^-2^ yr^-1^) suppressed biomass, mainly through suppressing the composition of annuals in the community (Zhou et al., 2018). Although research has concluded that an increase in N deposition would favor ephemeral composition in a temperate desert community, it is still difficult to know whether high levels of N have a positive or negative effect on growth and biomass allocation of fast-growing ephemerals and slow-growing annuals if growing separately rather than in a mixed community. Since plant growth and biomass allocation is closely related to N remaining in the plant–soil system and the N allocation between plant and soil pools (Templer et al., 2012), knowledge of biomass accumulation and allocation in desert plants under different N additions is crucial to quantifying ecosystem dynamics and ecosystem N fluxes.

Excess N addition can cause nutrient imbalance and reduce ecosystem productivity, once N supply exceeds the amount needed for plant growth (Homyak et al., 2014). A recent ^15^N labeled experiment showed that the total added N recovery of a *Haloxylon ammodendron* dominated desert ecosystem in the Gurbantunggut desert was on average 52%; ephemerals contained almost 86% of the N retained in the herbaceous layer (which included ephemerals, annuals and perennials) (Cui et al., 2017). Another study also demonstrated that ephemerals retained more N than summer annuals due to their relatively higher density and biomass in a temperate desert ecosystem (Huang et al., 2016). The higher ^15^N retention of ephemerals might be due to their higher capacity for N uptake or their domination in the community, or to their larger biomass than annuals. In N-limited grassland soils, fast-growing species can take up more N than slow-growing species (Harrison et al., 2008). Plant–soil ^15^N retention was found to be determined by the proportion of herbs, dominant plant traits, and the phenology of the plants by Mauritz et al. (2014) and De Vries and Bardgett (2016). However, the relationship of plant N retention and plant–soil recovery of N in desert plants with different growth strategies under certain N addition levels is not clear.

The Gurbantunggut Desert, located in Northwest China in Central Asia, is a typical temperate desert. Shrubs and densely distributed herbs are the dominant species (Angert et al., 2007). Ephemerals are the main species in the herbaceous layer (which includes ephemerals, ephemeroids, annuals and perennials), accounting for more than 80% of the plant biomass (Huang et al., 2016). Due to increased anthropogenic activities, especially farming, the area surrounding the Gurbantunggut Desert has received large amounts of N from atmospheric deposition (Li et al., 2012; Liu et al., 2013; Xu et al., 2015). We hypothesize that ephemerals and annuals will respond differently to this extra N because of their differing growth strategies. To test our hypotheses, we chose two of the most typical native species of Central Asian Desert ecosystems, *Malcolmia africana* and *Salsola affinis* to compare. *M. Africana* is a fast-growing and “opportunistic” ephemeral species; in contrast, *S. affinis* is a slow-growing and “conservative” annual species. We hypothesized that (1) high N addition would inhibit the growth and biomass accumulation of the ephemeral, and decrease the aboveground:belowground partitioning of N (the R:S ratio) of the ephemeral; (2) the ephemeral, with a shorter growing cycle and higher relative growth rate, would take up and retain significantly more N than the annual with its longer growing cycle; (3) the plant–soil recovery of N in the ephemeral would be significantly higher than that of the annual.

## Materials and Methods

### Plant Material

*Malcolmia Africana* is an ephemeral belonging to the family *Brassicaceae*, an “opportunistic” C3 species, whose germination, growth, flowering and fruiting and entire life cycle is highly dependent on precipitation and temperature (Yuan and Tang, 2010). In general, ephemerals can take advantage of water and available nutrients to complete their growing cycles before high temperature and drought can restrict growth, and reach peak biomass in late May (Yuan and Tang, 2009). *S. affinis* is an annual and “conservative” species belonging to the family *Chenopodiaceae.* It is a C4 species whose photosynthetic and water utilization pathway is more favorable for survival and growth and has evolved in plants under the stresses of high temperature and drought conditions (Su and Xie, 2011). Annuals generally have slower growth rates and a longer growing season (from March to October), with peak biomass in mid-August (Wang et al., 2006). Seeds of *M. africana* and *S. affinis* were collected in June and October in 2015, respectively, from the southern edge of the Gurbantunggut Desert. After air-drying, the seeds were stored at ambient temperature (20–25°C) until the experiment began in April 2016.

### Experimental Site and Design

The experiment was carried out at Fukang Station of Desert Ecology, Chinese Academy of Sciences, on the southern edge of the Gurbantunggut Desert (44^°^30′ N, 87^°^45′ E and 460 m a.s.l.). To compare the effect of N addition on the growth and N retention of ephemeral and annual herbs, a pot experiment was conducted, focusing on the two typical desert plant species described above. The experiment comprised five N rates [no N (N0), 15 kg N ha^-1^ (N15), 30 kg N ha^-1^ (N30), 60 kg N ha^-1^ (N60) and 120 kg N ha^-1^ (N120)] with ten replicates of each treatment. The N30 rate approximated to the current N deposition in the study area (35.4 kg N ha^-1^ yr^-1^) (Li et al., 2012; Song, 2015). N15 simulated a low N deposition; N60 and N120 higher depositions, following the prediction that global N deposition will double by 2050 relatively to the early 1990s (Galloway et al., 2008).

Surface soil (0–20 cm) from where the seeds were collected was used as the growth substrate. The soil samples were combined and thoroughly mixed. Soil physical and chemical properties are described in Supplementary Table S1. Fifty plastic pots (26 cm diameter and 19 cm depth) were filled with 8 kg soil for each species, and 20 seeds sown in each pot on 9 April 2016, maintaining the same seeding and germination schedule as in the field. All pots were placed in a controlled-environment greenhouse at Fukang Station at 30/11°C day/night temperature regime and a photosynthetic photo flux density of 1600 μmol^-1^ s^-1^ m^-2^ at midday. The relative air humidity ranged from 35 to 60%.

The pots were well watered with distilled water to achieve field capacity before the seeds sprouted. After establishment, some 25 days after sowing, the seedlings were thinned to 6 plants per pot to reduce competition for nutrients. The corresponding amount of ^15^NH_4_^15^NO_3_ fertilizer (5.16% abundance, Shanghai Research Institute of Chemical Industry) was calculated for each treatment based on the pot soil surface area and 0, 0.8, 1.6, 3.2, and 6.4 g N pot^-1^ added for the N0, N15, N30, N60, and N120 treatments, respectively. The fertilizer was dissolved in 100 mL distilled water and the solution applied once with an injector inserted into soil at 5 cm depth at the four corners of the pot. Soil in all pots was then maintained at 60–70% of field capacity and the pots moved randomly each week to minimize any effects of position.

### Sampling and Analysis

Six plants from each pot were harvested shortly after flowering (time of peak biomass) to measure plant growth and biomass allocation. The samplings were at 72 and 149 days after sowing *M. africana* and *S. affinis*, respectively, and were taken to represent the growing season length of the plants. Prior to harvest, the aboveground plant growth parameters including height, number of leaves and branches of each plant were recorded. The aboveground parts were then clipped with scissors and separated into stems and leaves. Following harvest, soil samples to 16.5 cm were carefully collected away from the taproot with a miniature auger (1 cm diameter). These samples were stored at <4°C in a refrigerator before analysis. All the soil samples from the pots were then washed with water to obtain coarse and fine roots, and the length of the longest root was measured and the number of lateral roots recorded. To calculate the biomass of stem, leaf, coarse roots and fine roots, all plant materials were oven-dried at 105°C for half an hour and then at 65°C for 48 h to a constant weight. After weighing, stems and leaves were mixed as the aboveground parts and ground to <0.25 mm in a high speed ball mill for analysis of total aboveground N content on an elemental analyzer (Vario Max CN, Elementar, Germany) and ^15^N abundance by mass spectrometry (Delta Plus XP, Thermo Finnigan, Pittsburg, PA, United States). The coarse and fine roots were combined as the belowground parts and analyzed in the same way. The fresh soil samples were air-dried, finely ground and sieved through <0.25 mm to measure total N content by elemental analysis as above and ^15^N abundance by mass spectrometry as above.

The percentage of N applied recovered in the aboveground and belowground parts of the plants and in the soil were calculated using the following equations (De Vries and Bardgett, 2016; Cui et al., 2017), with ^15^N atom% excess corrected for background abundance (0.3663%; Cabrera and Kissel, 1989).

(1) Applied N (Ndff) in the plant (kg N ha^-1^) = (plant N (g kg^-1^) × biomass (g pot^-1^) × 10^-2^/0.053 (m^2^ pot^-1^)) × ^15^N_plant_ atom% excess/^15^N_fertilizer_ atom% excess
(2) Applied N (Ndff) in soil (kg N ha^-1^) = (soil N (g kg^-1^) × soil weight (g pot^-1^) × 10^-2^/0.053 (m^2^ pot^-1^)) × ^15^N_soil_ atom% excess/^15^N_fertilizer_ atom% excess
(3) Fertilizer N recovery (%) = Ndff/^15^N rate × 100

### Statistical Analysis

The effects of N on plant growth characteristics, biomass production and allocation, N content, ^15^N abundance, N retention and recovery in *M. africana* and *S. affinis* were examined by two-way ANOVA with SAS V8 software (Version 8.0, SAS Institute Inc., Cary, NC, United States). A significant effect was determined as LSD at *P* < 0.05. Biomass and plant N retention response curves to N rates, and N input values at which maximum values of biomass and N retention occurred, were calculated using a quadratic-plateau model in R 3.3.1 (R Core Team, 2016) with packages “easyreg” and “er1” function (R Core Team, 2016) (Supplementary R code). Structural equation modeling (SEM) was used to test the direct and indirect effects of growing season length and N rate on ecosystem N retention (Supplementary R code). The model was constructed based on the theoretical knowledge and criterion described by De Vries and Bardgett (2016). SEM was performed with the “specifyModel” function in the “SEM” package of R 3.3.1 (R Core Team, 2016). All figures were drawn with Origin software 2015 (Origin Lab, Northampton, MA, United States).

## Results

### Plant Growth

**FIGURE 1:**
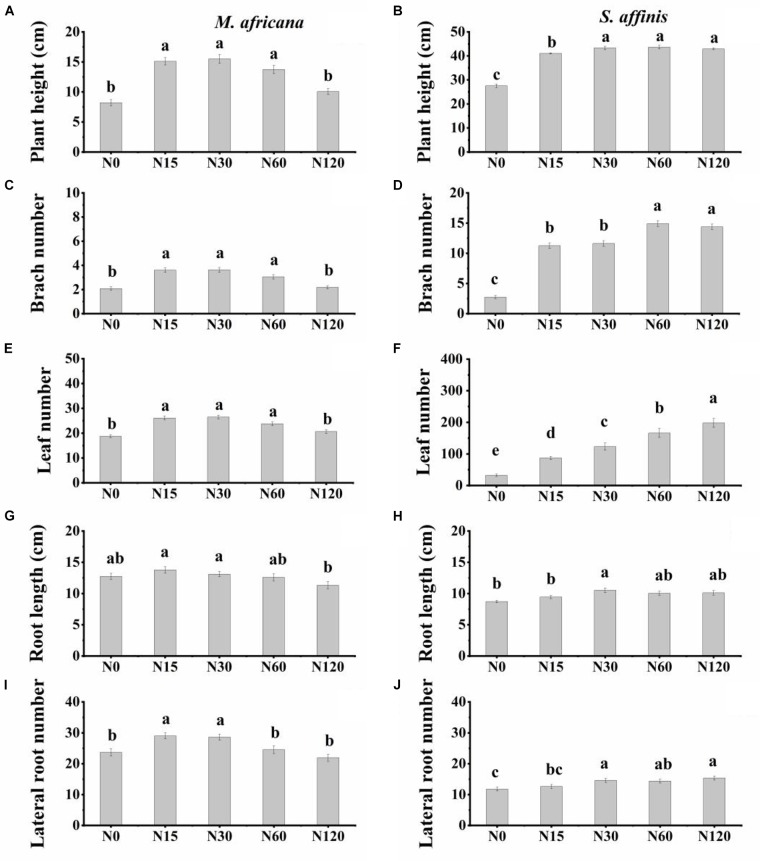
Plant height, number of branches, number of leaves, root length, and number of lateral roots of *M. africana* **(A,C,E,G,I)** and *S. affinis* **(B,D,F,H,J)** for each treatment. Bars represent means ± standard error (*n* = 10). Columns with different lowercase letters differ significantly at *P* = 0.05.

Plant height, branch number, leaf number, root length and lateral root number were significantly different between *M. africana* and *S. affinis* at each N rate (Table 1). Plant height, branch number and leaf number of *S. affinis* were significantly higher than those of *M. Africana*, whereas root length and lateral root number of *S. affinis* were significantly lower than those of *M. Africana* (Figure 1 and Table 1). For *M. Africana*, except for root length, low and moderate amounts of N (N15 and N30) significantly enhanced plant height, branch number, leaf number and lateral root number compared with the control, but high N rates (especially N120) significantly inhibited plant growth compared with low and moderate N rates (N15 and N30) (Figure 1A,C,E,G,I). For *S. affinis*, all plant growth parameters increased with N addition (Figure 1B,D,F,H,J).

**Table 1:** Two-way ANOVA analyses of species and N treatment on plant morphology and biomass partitioning.

Dependent variable	Independent variable	Df	*F*-value	*P*-value
Plant height	Species	1	4024.42	<0.0001
	Treatment	4	115.08	<0.0001
Branch number	Species	1	841.52	<0.0001
	Treatment	4	96.3	<0.0001
Leaf number	Species	1	832.26	<0.0001
	Treatment	4	13.57	<0.0001
Root length	Species	1	134.88	<0.0001
	Treatment	4	2.69	0.03
Lateral root number	Species	1	470.72	<0.0001
	Treatment	4	5.52	0.0002
Leave biomass	Species	1	608.64	<0.0001
	Treatment	4	42.57	<0.0001
Stem biomass	Species	1	568.84	<0.0001
	Treatment	4	35.92	<0.0001
Coarse root biomass	Species	1	28.13	<0.0001
	Treatment	4	27.53	<0.0001
Fine root biomass	Species	1	70.6	<0.0001
	Treatment	4	34.09	<0.0001
Aboveground biomass	Species	1	657.32	<0.0001
	Treatment	4	44.32	<0.0001
Belowground biomass	Species	1	73.16	<0.0001
	Treatment	4	35.85	<0.0001
R:S	Species	1	371.29	<0.0001
	Treatment	4	9.96	<0.0001


### Biomass and Biomass Partitioning

**FIGURE 2:**
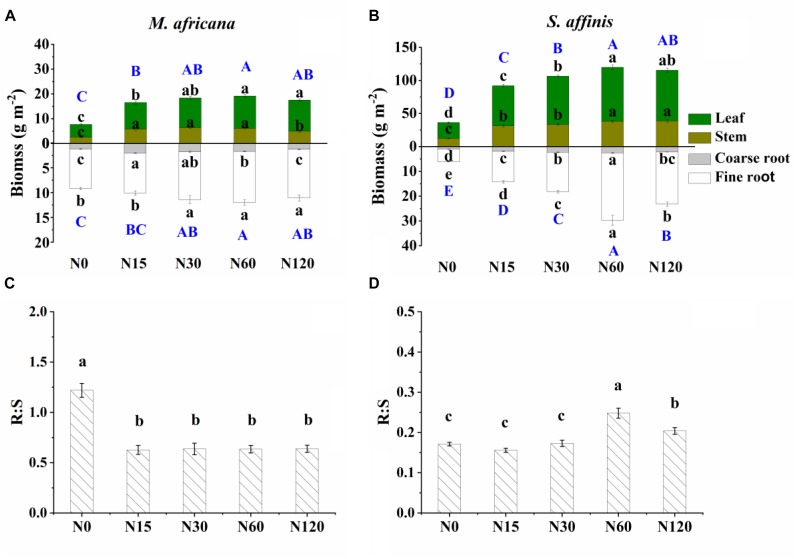
Biomass partitioning (including leaf biomass, shoot biomass, coarse root biomass and fine root biomass) and R:S ratio of *M. africana* **(A,C)** and *S. affinis* **(B,D)** at each N treatment. Bars represent means ± standard error (*n* = 10). Different lowercase letters above each column show values differ significantly at *P* = 0.05, and different uppercase letters (with blue color) indicate that the total aboveground biomass (the sum of leaf biomass and shoot biomass) or belowground biomass (the sum of coarse root biomass and fine root biomass) differ significantly at *P* = 0.05.

All measured plant biomass fractions of *S. affinis* were significantly higher than those of *M. Africana* (Table 1 and Figure 2). The mean aboveground biomass of *M. Africana* and *S. affinis* ranged from 7.7 to 19.1 g m^-2^, and from 36.2 to 115.4 g m^-2^, respectively. The mean belowground biomass of *M. Africana* and *S. affinis* ranged from 9.2 to 12.0 g m^-2^, and from 6.2 to 29.7 g m^-2^, respectively (Figure 2A,B). For *M. Africana*, low and moderate N addition (N15 and N30) significantly increased leaf, stem, fine root, coarse root biomass, aboveground biomass and belowground biomass; however, compared to the moderate N rate (N30), high N addition (especially N120) significantly inhibited stem and coarse root biomass (Figure 2A). For *S. affinis*, leaf, stem, coarse root, fine root, aboveground biomass and belowground biomass were all significantly enhanced by N addition (Figure 2B). The R:S ratio of *M. Africana* decreased significantly with N addition (Figure 2C); in contrast the R:S ratio of *S. affinis* increased with N addition (Figure 2D).

**FIGURE 3:**
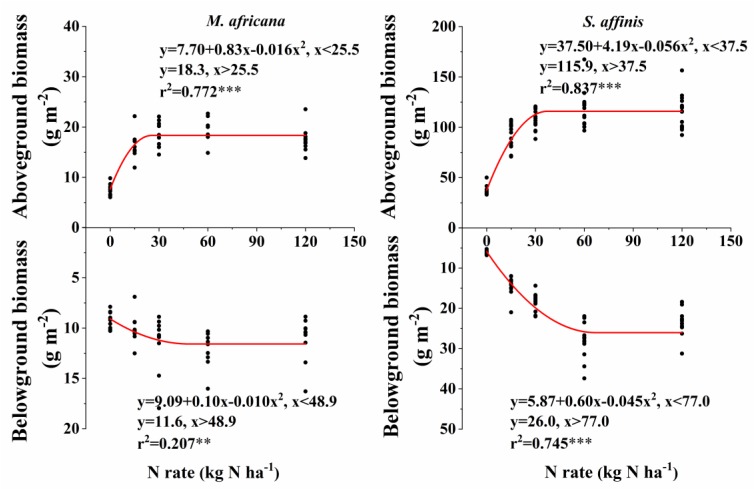
Responses of aboveground and belowground biomass of *M. africana* **(left)** and *S. affinis* **(right)** to increasing N addition. The asterisks on the panel represent the statistical results of the relationship, in which ^∗^, ^∗∗^, ^∗∗∗^, represent statistical significance at *P* < 0.05, *P* < 0.01, and *P* < 0.001, respectively.

Aboveground and belowground biomass N response curves are shown in Figure 3. All curves fitted the quadratic-plateau model well. The aboveground biomass and belowground biomass of *M. Africana* were positively correlated with N addition up to a threshold of 25.5 and 48.9 kg N ha^-1^ added, respectively. Similarly, the aboveground biomass and belowground biomass of *S. affinis* were also positively correlated with N addition up to significantly higher thresholds of 37.5 and 77.0 kg N ha^-1^ added, respectively. The maximum aboveground biomass and belowground biomass for *M. Africana* were 18.3 and 11.6 g m^-2^, and for *S. affinis* were 115.9 and 26.4 g m^-2^, respectively.

### N Retention Added ^15^N

**FIGURE 4:**
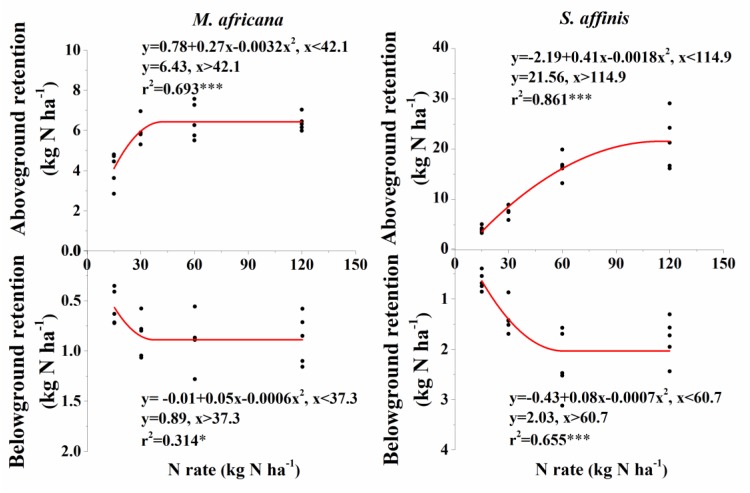
Aboveground and belowground ^15^N retention of *M. africana* **(left)** and *S. affinis* **(right)** in response to increasing N addition. The asterisks on the panel represent the statistical results of the relationship, in which ^∗^, ^∗∗^, ^∗∗∗^, represent statistical significance at *P* < 0.05, *P* < 0.01, and *P* < 0.001, respectively.

ANOVA analysis showed that ^15^N retention in the aboveground and belowground parts of the plants, in the soil and that lost, significantly differed between *M. Africana* and *S. affinis* as well as between treatments (Table 2). Except at low N addition (N15), aboveground and belowground retentions of ^15^N in *S. affinis* were significantly higher than those in *M. Africana*. Response curves of aboveground and belowground ^15^N retention of *M. Africana* and *S. affinis* are illustrated in Figure 4, showing a good fit to the quadratic-plateau model. For *M. Africana*, the relationships between aboveground retention and belowground ^15^N retention and N addition had maxima of 6.4 and 0.9 kg N ha^-1^, respectively, at N additions of 42.1 and 37.3 kg N ha^-1^, respectively. Similarly, *S. affinis* had maximum ^15^N retentions of 21.6 and 2.0 kg N ha^-1^ in aboveground and belowground parts at N applications of 114.9 and 21.6 kg N ha^-1^, respectively. *M. Africana* retained significantly more N in its aboveground and belowground components under moderate and high N additions than under low N addition. However, there were no significant differences between the N30, N60 and N120 treatments. ^15^N retained in the soil and lost from the plant–soil system of *M. Africana* increased significantly with increasing N applied (Table 2). For *S. affinis*, N retention in the aboveground and belowground components of the plant, in the soil, and the amount lost, significantly increased with increasing N rate (Table 2).

**Table 2:** Fate of ^15^N applied to the plant–soil system of *M. africana* (ephemeral) and *S. affinis* (annual), and two-way ANOVA analyses of the effects of species and N treatment on ^15^N partitioning in the plant–soil system.

Species	Treatment	^15^N addition (kg ha^-1^)	Aboveground (kg ha^-1^)	Belowground (kg ha^-1^)	Soil (kg ha^-1^)	Loss (kg ha^-1^)
*M.* *africana*	N15	15	4.09 ± 0.37b	0.57 ± 0.08b	7.87 ± 0.9c	2.47 ± 0.58c
	N30	30	5.96 ± 0.27a	0.85 ± 0.09 ab	13.85 ± 1.38c	9.33 ± 1.38bc
	N60	60	6.47 ± 0.41a	0.9 ± 0.12a	33.92 ± 3.68b	18.71 ± 3.65b
	N120	120	6.39 ± 0.18a	0.88 ± 0.11a	63.51 ± 6.94a	49.23 ± 7.12a
*S. affinis*	N15	15	4.06 ± 0.29d	0.65 ± 0.08c	4.52 ± 0.24c	5.78 ± 0.31d
	N30	30	7.79 ± 0.56c	1.39 ± 0.14b	6.7 ± 0.62c	14.13 ± 1.05c
	N60	60	16.55 ± 1.06b	2.28 ± 0.29a	11.31 ± 0.5b	29.86 ± 1.41b
	N120	120	21.5 ± 2.41a	1.8 ± 0.19 ab	19.91 ± 1.77a	76.8 ± 3.94a
Source (*P*-values)	Species		<0.0001	<0.0001	<0.0001	<0.0001
	Treatment		<0.0001	<0.0001	<0.0001	<0.0001


**FIGURE 5:**
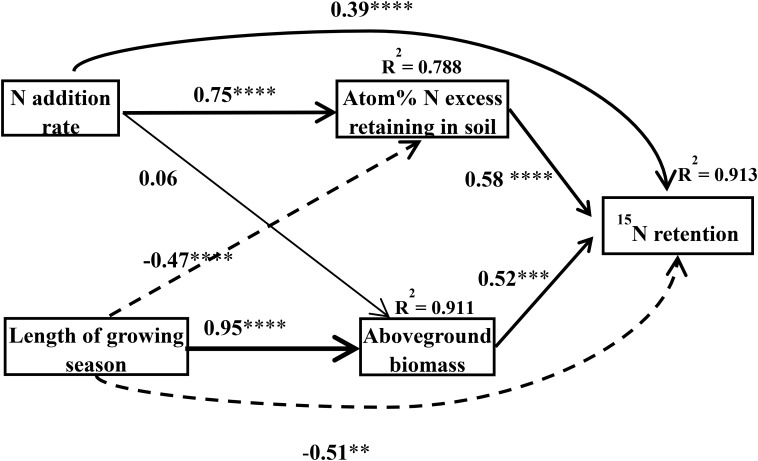
Final structural equation models describing the effects of the N treatment and the length of growing season on N retention in the plant–soil system as separated into aboveground biomass and atom% ^15^N in the soil. Boxes contain measured variables. Arrows indicate the hypothesized causal relationship (paths), and the width of the arrow denotes the strength of the relationship. The number and asterisks on the arrows represent the path coefficients and the statistical results of the relationship, in which ^∗^, ^∗∗^, ^∗∗∗^, ^∗∗∗∗^ represent statistical significance at *P* < 0.05, *P* < 0.01, *P* < 0.001, *P* < 0.0001, respectively. Solid arrows represent positive effects, dashed arrows represent negative effects. *R*^2^ values represent the proportion of variance explained by the model for the response variables. This model fitted well [χ^2^ = 1.57, Df = 2, Pr (>Chisq) = 0.457, AIC = 27.6, BIC = -5.81].

Application of the SEM suggested that the plant–soil ^15^N retention was directly and indirectly affected by N rate and the length of the growing season of the plants [χ^2^ = 1.57, Df = 2, Pr (>χ^2^) = 0.457, AIC = 27.6, BIC = -5.81] (Figure 5). Nitrogen addition can directly and indirectly affect plant–soil ^15^N retention through the ^15^N excess that remains in the soil (the standardized indirect effect of excess ^15^N remaining in the soil = 0.75 × 0.58 = 0.435). In addition, the length of the growing season can also directly and indirectly affect plant–soil ^15^N retention through the aboveground biomass (the standardized indirect effect of aboveground biomass on ecosystem ^15^N retention = 0.95 × 0.52 = 0.494), and the ^15^N remaining in the soil (the standardized indirect effect of ^15^N excess remaining in the soil on ecosystem ^15^N retention = -0.47 × 0.58 = -0.273). In general, the N addition rate was positively related to plant–soil ^15^N retention, whereas the length of growing season was negatively related to plant–soil ^15^N retention.

### N Recovery of Added ^15^N

**FIGURE 6:**
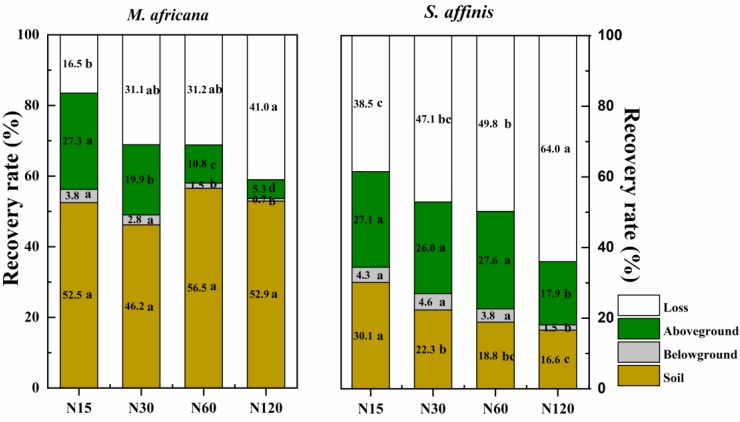
The percentage of added ^15^N recovered in the soil, the belowground and aboveground parts of the plants, and that lost to air or water in *M. africana* **(left)** and *S. affinis* **(right)** under the different N treatments. Values with different letters differ significantly between treatments at *P* = 0.05.

The mean ^15^N recovery in the *M. Africana* and *S. affinis* plant–soil systems was 70.1 and 50.2%, respectively. For *M. Africana*, most of the ^15^N was retained in the soil or lost, whereas for *S. affinis* most of the ^15^N was in the aboveground part of the plants or lost (Figure 6). The recovery of ^15^N in the soil under *M. Africana* was 52.0%, significantly higher than that of *S. affinis.* In contrast, the recovery of ^15^N in the aboveground and belowground parts of the plant and the loss from *M. Africana* was significantly lower than that of *S. affinis*. Recovery in the whole plant–soil system of *M. Africana* was significantly higher than that of *S. affinis* at the same N rate. Except for retention in the soil under *M. Africana*, the recovery of ^15^N in the different components of the two plant–soil systems significantly decreased with increasing ^15^N addition, whereas losses significantly increased with ^15^N addition.

## Discussion

### Growth and Biomass Responses to N Addition

In arid and semi-arid ecosystems, water availability is the main factor limiting plant growth, community composition and community productivity (Yahdjian and Sala, 2006; Miranda et al., 2009; Robertson et al., 2009). Nitrogen can become the main limiting factor if drought stress is alleviated, and extra N combined with enough water can then promote plant growth and increase community productivity (Ladwig et al., 2012; Fan et al., 2013). Our study confirmed this in both the ephemeral and annual plants, as the pot experiment was conducted with adequate water. However, the effect of N on the growth and biomass of the ephemeral and annual differed. High N addition had little effect or even inhibited plant growth and biomass accumulation of *M. Africana* compared to *S. affinis*.Wu et al. (2008) reported that adding large amounts of N did not affect the height, basal diameter, leaf number, leaf area and biomass of *Sophora davidii* (a shrub) seedlings, but root length decreased with increasing N supply. Moderate amounts of extra N promoted aboveground growth and root development, but excess N decreased root growth or even damaged roots (Salih et al., 2005; Wu et al., 2008). Similarly, the response curves also provided direct evidence of the different response of the two species to N addition (Figure 3). The N rate at which maximum growth of the ephemeral was observed was significantly less than that of the annual, which indicates that the ephemeral was more sensitive to N than the annual. Similar results were found between Japanese red pine and Japanese cedar: generally species grown in nutrient-poor habitats were more sensitive to high N deposition and its growth were significantly reduced under the highest N treatment (Nakaji et al., 2001).

The allocation of biomass between aboveground and belowground components of plants (the R:S ratio) is determined by species, ontogenetic development and environmental change (Poorter and Nagel, 2000). We found that the R:S ratio of the ephemeral was significantly higher than that of the annual. This difference was most likely due to the phenological differences and the relative ability to adapt to growing conditions, i.e., the environment. We found that the R:S ratio of the ephemeral decreased with N input in accordance with the “functional equilibrium” model (Poorter and Nagel, 2000; Poorter et al., 2012), consistent with our original hypothesis. However, the response of the R:S ratio of the annual plant to N addition did not fit with the “functional equilibrium” model. In a study of 27 herbaceous species, Muller et al. (2000) found that the R:S ratio of “opportunistic” species fitted the general relationship, decreasing with nutrient input, whereas “conservative” species from nutrient-poor habitats increased their R:S ratio under higher nutrient inputs. Similarly, in the current study, the ephemeral is an “opportunistic” and resource-exploitative species whose adaptive capacity was less than that of “conservative” and resource-conservative species. Therefore, the growth of the ephemeral was inhibited under high N addition. Adaptive responses in physiology and morphology may be the main mechanism by which different species deal with environmental change in their habitats (Patterson et al., 1997; Poorter et al., 2012). The positive response of the R:S ratio of *S. affinis* to N also supported our finding that the root morphology of the annual could adapt to high N addition by increasing the number of lateral roots as N input increased.

### Response of Plant–Soil ^15^N Retention to N Addition Rate

Added N significantly increased ^15^N retention in aboveground and belowground parts of the plant, but this varied between the species. In N-limited ecosystems, grassland species with different growth strategies also showed species-specific differences in the amount of N retained. Fast-growing and resource-exploitative species usually retained more N than slow-growing and resource-conservative species (Bardgett et al., 2003; Weigelt et al., 2005; Harrison et al., 2007, 2008; Grassein et al., 2015; De Vries and Bardgett, 2016). However, contrary to our original hypothesis, the difference in ^15^N retention between the two species was dependent on the amount of ^15^N added. It has been suggested that the species-specific ^15^N retention in plants is related to the plant relative growth rate (Weigelt et al., 2005). Alternatively, it has been pointed out that plant traits rather than plant growth rate or trait functional diversity could determine the inter-specific plant ^15^N retention (Harrison et al., 2008; De Vries and Bardgett, 2016). In our experiment, especially in the low N treatment, although the aboveground and belowground biomass of *M. Africana* were significantly less than that of *S. affinis* (Figure 2A,B; *P* < 0.0001; *P* < 0.0001), we did not find significant differences in the ^15^N retained in the aboveground and belowground components between *M. Africana* and *S. affinis* due to the higher aboveground and belowground ^15^N uptake of *M. Africana* (Supplementary Figures S1A,B). Under moderate and high N additions, both the ability to take up ^15^N and the growth of the ephemeral aboveground and belowground were suppressed, causing ^15^N retention in the aboveground and belowground parts of the ephemeral to be less than those of the annual (Figure 2 and Supplementary Figure S1). Therefore, it can be inferred that the higher retention of N by the ephemeral in the community compared to that of the annual in the field experiment of Cui et al. (2017) was not due to the higher uptake ability of ^15^N of the ephemeral under moderate (N30) and high N (N60) addition. Clearly, species differences in the N-retention of plants at different N rates can be explained by plant growth, shoot and root uptake capacity.

The SEM results also showed that N rate and the length of the growth season can directly affect N retention in the whole plant–soil system, and also indirectly affect it through the aboveground biomass and the amount of excess N that remains in the soil (Figure 5). De Vries and Bardgett (2016) pointed out that root biomass and the dominant plant trait, and the amount of N retained in the soil, can control short-term ecosystem N retention. Aboveground biomass was positively related to belowground biomass, and the amount of ^15^N retained in the soil was related to the ability of both the aboveground and belowground components to take up ^15^N. Therefore, aboveground biomass and the amount of excess ^15^N retained in the soil was used to construct the SEM and describe the whole plant–soil system ^15^N retention. Thus, although the plant N retention by the ephemeral was significantly less than that of the annual, the plant–soil retention of the annual was significantly higher than that of the ephemeral due to the high retention of ^15^N in the soil.

### Plant–Soil ^15^N Recovery

Consistent with our third hypothesis mentioned above, the average recovery rate of ^15^N by the ephemeral and annual plant–soil systems were 70 and 50%, respectively. In previous research, the recovery of ^15^N in a *Haloxylon ammodendron* dominant temperate desert ecosystem was approximately 52% on average, in which the soil and the plant accounted for about 40 and 12% of the total recovery, respectively (Cui et al., 2017). The higher recovery rate in the pot experiment compared to the field experiment could be due to the lack of drought stress and competition, and plant community differences, and of course the more effective exploitation of the soil by plant roots. Although the annual had a higher plant N recovery rate than the ephemeral, the whole plant–soil system N recovery of the annual was significantly less than that of the ephemeral. Deserts with coarse-textured soils usually have a lower water-holding capacity and labile C and N pools, and slower N mineralization and immobilization than other ecosystems (Austin et al., 2004). Increased inputs of N to coarse desert soils dominated by long growth period annuals is therefore likely to result in less N being retained in the soil and a higher risk of N loss to the environment (air or water or both) compared with the same system with shorter growth period ephemerals, especially in the artificial confines of a pot experiment maintained at 60–70% field capacity (Supplementary Figure S1C). This was confirmed by the negative relationship in the SEM between the atom% ^15^N excess remaining in the soil and the growing period of the plants (Figure 5).

Increasing N inputs are likely to significantly decrease total ecosystem N recovery in both ephemeral and annual plant–soil systems. Consistent with this, a meta-analysis of natural terrestrial ecosystems showed that N recovery was negatively related to N addition when inputs exceeded 46 kg N ha^-1^ yr^-1^ (Templer et al., 2012). Similarly, Cui et al. (2017) concluded that N recovery significantly decreased when N deposition increased from 30 kg N ha^-1^ yr^-1^ to 60 kg N ha^-1^ yr^-1^ in a temperate desert ecosystem in Northwest China. Due to the poor nutrient content and dry conditions, plant coverage is sparse in desert ecosystems and microbial community sizes are small, so excessive N inputs could easily exceed the biotic demands and cause ecosystem nutrient imbalances and thus increased gaseous losses or leaching or both (McCalley and Sparks, 2008, 2009; Homyak et al., 2014). Zhou et al. (2018) pointed out that the responses to N of the community structure, richness, evenness, density and biomass of herbaceous plants were clearly N rate-dependent, with N addition increasingly selecting nitrophilic, fast-growing species rather than slower growing species. Therefore, under enhanced N deposition (Liu et al., 2013) and future climate scenarios, the N recovery of desert ecosystems may vary substantially and show its species dependence with change of community composition.

## Conclusion

Our study has shown clear differences in plant growth, allocation and plant–soil system N recovery responses to increasing N addition for two typical temperate desert species, *M. Africana* (an ephemeral) and *S. affinis* (an annual). Low and moderate N additions significantly enhanced plant growth and biomass production of both the ephemeral and annual, whereas high N addition inhibited plant growth and biomass of the ephemeral but not the annual. The amount of N applied at which the maximum retention of N in the aboveground and belowground biomass of the ephemeral was significantly less than that of the annual. In addition, except at low N addition, the plant N retention of the ephemeral was significantly less than that of the annual, but the total plant–soil retention was significantly greater. The whole plant–soil ecosystem N recovery will therefore decrease with predicted increases in future N deposition. The results indicate that N recovery of this temperate desert ecosystem is likely to vary as species composition of the community also changes with future climate change and enhance N deposition.

## Author Contributions

XL designed the experiments. XC, PY, WW, YG, and KL conducted the experiments. XC, XL, KG, and TM wrote the manuscript. All authors reviewed and commented on the manuscript.

## Conflict of Interest Statement

The authors declare that the research was conducted in the absence of any commercial or financial relationships that could be construed as a potential conflict of interest. The reviewer XZ declared a shared affiliation, with no collaboration, with several of the authors, PY, WW, YG, and KL, to the handling Editor at the time of review.
