# Supplementary material for: The Growth and N Retention of Two Annual Desert Plants Varied Under Different Nitrogen Deposition Rates
Source: Front Plant Sci. 2019 Mar 26;10:356. doi: 10.3389/fpls.2019.00356 (PMC6443888; doi:10.3389/fpls.2019.00356)
Supplement: Supplementary file 1 [file Table_1.docx]

**Supporting Materials of**

**Two annual desert plants varying in plant growth and nitrogen retention under different nitrogen deposition rates**

Xiaoqing Cui^1,2†^, Ping Yue^3,4†^, Wenchao Wu^3,5^, Yanming Gong^3^, Kaihui Li^3^, Tom Misselbrook^6^, Keith Goulding^7^, Xuejun Liu^1*^

^1^Key Laboratory of Plant-Soil Interactions of MOE, Beijing Key Laboratory of Farmland Soil Pollution Prevention and Remediation, College of Resources and Environmental Sciences, China Agricultural University, Beijing, China

^2^Sino-France Institute of Earth Systems Science, Laboratory for Earth Surface Processes, College of Urban and Environmental Sciences, Peking University, Beijing, 100871, China

^3^State Key Laboratory of Desert and Oasis Ecology, Xinjiang Institute of Ecology and Geography, Chinese Academy of Sciences, Urumqi, China

^4^Urat Desert-Grassland Research Station, Northwest Institute of Eco-Environment and Resources, Chinese Academy of Sciences, Lanzhou 730000, China

^5^College of Life Science, University of the Chinese Academy of Sciences, Beijing, China

^6^The Sustainable Soils and Grassland Systems Department, Rothamsted Research, North Wyke, Devon, UK

^7^The Sustainable Soils and Grassland Systems Department, Rothamsted Research, Harpenden, UK

† These authors contributed equally to this work co-first authors

^*^ Correspondence to: Xuejun Liu

Address: China Agricultural University, Beijing 100193, China

Email address: [liu310@cau.edu.cn](mailto:liu310@cau.edu.cn)

Telephone number: +86 (0) 10 62733459

**Supplementary Figure legends**

**FIGURE S1.** N content (open bars) and atom% ^15^N excess (diagonal bars) of the *M. africana* (white) and *S. affinis* (gray) plant-soil systems including aboveground components (a), belowground components (b) and the soil (c) at each ^15^N rate. Bars represent means ± standard error (n=5); ns represents no significant difference between *M. africana* and *S. affinis*, *, **, ***, **** represent significant difference between *M. africana* and *S. affinis* at *P*<0.05, *P*<0.01, *P*<0.001, *P*<0.0001, respectively.

FIGURE S1





Supplementary Table legends

TABLE S1 Physical and chemical properties of the soil used in the pot experiment

TABLE S1

| Soil properties | pH | SOC  (g kg^-1^) | TN  (g kg^-1^) | C:N | Nmin  (kg ha^-1^) | Olsen-P  (mg kg^-1^) | Soluble K  (mg kg^-1^) |
| --- | --- | --- | --- | --- | --- | --- | --- |
| mean ± SE | 8.91±0.08 | 1.30±0.12 | 0.04±0.001 | 32.86±2.56 | 3.68±0.96 | 7.47±0.37 | 125.7±2.62 |

Supplementary R code

#####R code for SEM ######

install.packages(“sem”) # installation package

install.packages(“dplyr”) # installation package

library(sem) # loading packages

library(dplyr) #loading packages

dat <-read.csv("semdata.csv",header=T) # loading data for constructing the structural equation model

dat

colnames(dat) <- c('a', 'b', 'c', 'd',"e") # assigning a new and simple name for each column

dat

cor_num <- cor(dat) # Compute correlation matrix

cor_num

model.kerch <- specifyModel(

text = '

a -> c, a_c, NA

a -> d, a_d, NA

a -> e, a_e, NA

b -> c, b_c, NA

b -> d, b_d, NA

b -> e, b_e, NA

c -> e, c_e, NA

d -> e, d_e, NA

a <-> a, a_a, NA

b <-> b, b_b, NA

c <-> c, c_c, NA

d <-> d, d_d, NA

e <-> e, e_e, NA

')

# Building a model. we assume that the variable “a” can affect “e” through “c” and “d”, and the variable “b” can also affect “e” through “c” and “d”. The SEM package submits a path relationship in text form and, as default, all variables are observed variables. The first column indicates the path and the second column indicates the regression coefficient. An arbitrary variable is assumed first, and the model will then perform the modified assignment. The third column is the starting value of the free parameter. If there is no value available, this is set to be NA, and the starting value calculated in the SEM package. #The last four lines represent the effects of the variables themselves.

# The second parameter can use the correlation matrix directly, or the covariance matrix

out_sem <- sem(model.kerch, cor_num, nrow(dat))

coef <- out_sem$coeff # obtaining regression coefficients

coeff_name <- out_sem$semmod[,1] # obtaining regression coefficient names

summary(out_sem) # obtaining the summary of sem

pathDiagram(out_sem, edge.labels="values",same.rank="c, d", edge.weight = "proportional",standardize =T,rank.direction="LR") # drawing the sem diagram

#####R code for quadratic-plateau model#####

install.packages("easyreg") # installation package

library(easyreg) # loading packages

data1<- read.csv("S.csv") # reading data of *Salsola affinis* aboveground biomass for construct model

er1(data1, model=4) # performing analysis of regression and calculation the regression parameters

regplot(data1, model=4,ylab="biomass", xlab="treatment") # ploting data and equation

library(easyreg)

data1<- read.csv("R.csv")

#reading data of *Salsola affinis* belowground biomass for constructing model

er1(data1, model=4)

regplot(data1, model=4,ylab="biomass", xlab="treatment")

library(easyreg)

data1<- read.csv("S1.csv")

#reading data of *Malcolmia* *Africana* aboveground biomass for constructing model

er1(data1, model=4)

regplot(data1, model=4,ylab="biomass", xlab="treatment")

library(easyreg)

data1<- read.csv("R1.csv")

#reading data of *Malcolmia* *Africana* belowground biomass for constructing model

er1(data1, model=4)

regplot(data1, model=4,ylab="biomass", xlab="treatment")

library(easyreg)

data1<- read.csv("s retention.csv")

# reading data of *Salsola affinis* aboveground ^15^N retention for constructing model

er1(data1, model=4)

regplot(data1, model=4,ylab="retention", xlab="treatment")

library(easyreg)

data1<- read.csv("r retention.csv")

# reading data of *Salsola affinis* belowground ^15^N retention for constructing model

er1(data1, model=4)

regplot(data1, model=4,ylab="retention", xlab="treatment")

library(easyreg)

data1<- read.csv("s retention1.csv")

#reading data of *Malcolmia* *Africana* aboveground ^15^N retention for constructing model

er1(data1, model=4)

regplot(data1, model=4,ylab="retention", xlab="treatment")

library(easyreg)

data1<- read.csv("r retention1.csv")

#reading data of *Malcolmia* *Africana* belowground ^15^N retention for constructing model

er1(data1, model=4)

regplot(data1, model=4,ylab="retention", xlab="treatment")
